# Comprehensive Analysis of WUSCEL-Related Homeobox Gene Family in Ramie (*Boehmeria nivea*) Indicates Its Potential Role in Adventitious Root Development

**DOI:** 10.3390/biology12121475

**Published:** 2023-11-28

**Authors:** Aminu Shehu Abubakar, Yongmei Wu, Fengming Chen, Aiguo Zhu, Ping Chen, Kunmei Chen, Xiaojun Qiu, Xiaoyu Huang, Haohan Zhao, Jikang Chen, Gang Gao

**Affiliations:** 1 Hunan Provincial Key Laboratory of the TCM Agricultural Biogenomics, Changsha Medical University, Changsha 410219, China; aashehu.agr@buk.edu.ng (A.S.A.); cfming@stu.hunau.cn (F.C.); 2 Institute of Bast Fiber Crops, Chinese Academy of Agricultural Sciences, Changsha 410221, China; 3 Department of Agronomy, Bayero University Kano, PMB 3011, Kano 700241, Nigeria; 4 National Nanfan Research Institute (Sanya), Chinese Academy of Agricultural Sciences, Sanya 572024, China; 5 Key Laboratory of Biological and Processing for Bast Fiber Crops, Changsha 410221, China

**Keywords:** WOX genes, ramie, adventitious roots, cuttings, segmental duplication, phylogenetic analysis, expression analysis

## Abstract

**Simple Summary:**

Adventitious root formation is a significant limiting factor in the vegetative propagation of economically important plants. Owing to the low germination rates and survival of conventional seed propagation, vegetative propagation remains the best option for large-scale production. Generally, there is a lack of competency for elite plant species cuttings or explants to form adventitious roots. The molecular-level studies of the adventitious root formation have not been explored in many species, such as ramie. As the WUSCEL-related homeobox gene family plays a critical role in promoting vegetative organs and stem cell functioning, we investigated their potential role in adventitious root formation in ramie using genome-wide characterization, gene structure, and expression analysis. The overall result indicated their possible involvement in rooting ramie cuttings. This study thus increases our understanding of the role of these genes and lays a foundation for further studies.

**Abstract:**

A WUSCHEL-related homeobox (WOX) gene family has been implicated in promoting vegetative organs to embryonic transition and maintaining plant embryonic stem cell identity. Using genome-wide analysis, we identified 17 candidates, WOX genes in ramie (*Boehmeria nivea*). The genes (BnWOX) showed highly conserved homeodomain regions typical of WOX. Based on phylogenetic analysis, they were classified into three distinct groups: modern, intermediate, and ancient clades. The genes displayed 65% and 35% collinearities with their *Arabidopsis thaliana* and *Oryza sativa* ortholog, respectively, and exhibited similar motifs, suggesting similar functions. Furthermore, four segmental duplications (*BnWOX10/14*, *BnWOX13A/13B*, *BnWOX9A/9B*, and *BnWOX6A/Maker00021031*) and a tandem-duplicated pair (*BnWOX5/7*) among the putative ramie WOX genes were obtained, suggesting that whole-genome duplication (WGD) played a role in WOX gene expansion. Expression profiling analysis of the genes in the bud, leaf, stem, and root of the stem cuttings revealed higher expression levels of *BnWOX10* and *BnWOX14* in the stem and root and lower in the leaf consistent with the qRT-PCR analysis, suggesting their direct roles in ramie root formation. Analysis of the rooting characteristics and expression in the stem cuttings of sixty-seven different ramie genetic resources showed a possible involvement of *BnWOX14* in the adventitious rooting of ramie. Thus, this study provides valuable information on ramie WOX genes and lays the foundation for further research.

## 1. Introduction

Ramie (*Boehmeria nivea* (L.) Gaud.) is a perennial bast fiber plant cultivated widely in southeastern Asia and the Pacific Rim, including China and India. Ramie is rich in protein and has higher biomass, making it a significant source of forage and quality fiber for textile industries [1]. The low germination rates and survival of conventional ramie seed propagation make vegetative propagation, such as hydroponic technology, the best option for large-scale production [2]. Due to a high degree of heterozygosity in ramie, vegetative propagation can increase its genetic stability and produce many clones within a short time, sustaining the increasing demand for raw ramie materials [3]. The availability of stable planting materials is critical in sustaining production and ensures profitable production and significant motivation to producers [4]. However, in many economically important plants, the formation of adventitious roots is a limiting factor for vegetative reproduction, and often, there is routinely a lack of competency for elite plant species cuttings or explants to form adventitious roots [5,6,7].

Adventitious root formation is an intrinsic component of both dicots and monocots triggered by various environmental and physiological cues such as wounding, nutrient deprivation, and flooding [5,8,9]. Roots (primary, lateral, or adventitious) represent a sophisticated plant system that helps them cope with environmental stresses and are essential channels between substrates and plant systems for the exchange of materials and anchorage [8,10]. The distinguishing feature of the adventitious root is that it is postembryonic and always develops from cells close to the vascular tissues as either part of the plant development program or in response to induction by exogenous application of hormones or wounding [5]. Adventitious rooting is controlled by endogenous and exogenous factors, with auxin (one of the key endogenous factors) reported as playing a significant role [11]. Adventitious roots are economically important in the propagation of cuttings of many industrial crops like ramie and ecologically contribute to the geomorphological stability of mangrove soils and tolerance to heavy metal stresses [9,12]. As a limiting factor in clonal propagation, rapid plant growth, and forage production, understanding the genetic mechanism and regulatory elements controlling adventitious rooting facilitates breeding to enhance ramie’s growth and development. In vascular plants, adventitious roots and all other organs are initiated from stem cells, and the WUSCHEL-related homeobox gene (WOX) has been implicated in stem cell functions and in promoting vegetative organs to embryonic transition and maintaining embryonic stem cell identity [13,14].

WOXs are transcription factor family genes distinguished by the presence of a homeodomain, which comprises 40–60 amino acid residues encoded by the homeobox (HB). They are expressed in the meristem organizing center located below the stem apical meristem stem cells and play a significant role during organogenesis, determining stem cell fate [15]. They regulate plant development and growth, partaking in cell dedifferentiation via the recruitment of transcriptional corepressors, which repress the genes that promote differentiation and ensure the maintenance of stem cells [16,17]. Many WOX gene family members were shown to partake in plant development [16]. *Arabidopsis thaliana WOX9* (*AtWOX9*), for example, is involved in aerial organs, roots, and vegetative shoot apical meristem growth [18]. In transgenic *Populus trichocarpa*, however, overexpression of *PtWOX13* promoted secondary cell wall thickening, enhanced fiber length, and increased lignin and hemicellulose content [19]. Functional losses of *AtWOX5* in the root stem cell due to mutation lead to terminal differentiation of the columella stem cell [20]. *AtWOX13*, which is dynamically expressed during the developmental processes of both primary and lateral roots, is involved in the floral transition. *AtWOX14* mutant resulted in premature anther differentiation, delayed floral shift, and abnormal root [15]. Recent findings confirmed the role of WOX genes in rooting and suggested its involvement in stress response, as overexpression of poplar *PagWOX11/12a* was reported to have increased root biomass and enhanced drought tolerance [21].

In previous studies, a variable number of WOX genes were reported in different plant species, such as 15 in *A. thaliana*, 13 in rice, 43 in bread wheat, 34 in *Medicago sativa*, 10 in *Jatropha curcas*, and 20 in *Hevea brasiliensis* [22,23,24,25]. Specific expression of the genes in various organs and cells in plants pointed to their crucial roles in organogenesis [15,26,27,28]. Despite these transcription family genes’ significance, they remain largely unexplored in ramie. The availability of the complete genome sequence of ramie will facilitate exploration and understanding of the molecular mechanism of the critical gene regulatory network like WOX. We therefore carried out a genome-wide characterization of the ramie WOX gene family; conducted a phylogenetic analysis with annotated homologs from *A. thaliana*, *P. trichocarpa*, cotton, and rice; performed chromosomal mapping of the identified members; and analyzed their functions and structure. We used transcriptome data of different bud, leaf, stem, and root of stem cuttings of ramie and quantitative real-time PCR (qRT-PCR) to investigate their involvement in adventitious root formation. We analyzed the rooting characteristics of stem cuttings in 67 elite ramie genetic resources to obtain insight into the role of the gene family in regulating adventitious roots.

## 2. Materials and Methods

### 2.1. Identification of WOX Genes in Ramie

The ramie WOX genes were identified from *B. nivea* genomic data (NCBI GenBank Accession number: GCA_002806895.1). Arabidopsis WOX protein sequences (https://arabidopsis.org, accessed on 29 April 2022) were used to filter the target with Blast GUI Wrapper in TBtools [29] with a cutoff e-value 1 × 10^−5^. The resulting candidate genes obtained were screened using the Conserved Domain Database [30], pfam [31], and SMART [32] to ensure the presence of a complete WOX domain. The putative genes were named *BnWUS*, *BnWOX1* to *BnWOX14* based on their evolutionary relationships with their annotated homolog in *A. thaliana* and rice and BLASTP search following previous reports [23,25,33,34]. Physical and chemical properties, including the number of amino acids, molecular weight, and grand average of hydropathicity were computed using the ExPasy online tool (http://expasy.ch/tool/protparam.html, accessed on 4 July 2022). The subcellular location of the WOX genes was predicted using the ProtComp v. 9.0 (http://www.softberry.com/berry.phtml, accessed on 12 July 2022) and CELLO v.2.5 subcellular localization prediction tools.

### 2.2. Multiple Sequence Alignment and Phylogenetic Analysis

Multiple sequence alignment using the protein sequences of the WOX genes was performed using ClustalW in MEGA X [35] version 10.1.8, and the result was visualized using Jalview [36]. Sequence logos were established using the WebLogo online tool [37].

Phylogenetic analysis was conducted using the *B. nivea* WOX protein sequences and those from other four species comprising *A. thaliana*, rice, *P.trichocarpa*, and *Gossypium aboreum* (Table S1). The phylogenetic tree was constructed using the neighbor-joining method [23] with the parameters Poisson model, gamma distributed (G), pairwise deletion, and 1000 bootstrap in MEGA X, and further confirmed using the maximum likelihood method [17,33] with the parameters LG Model, G + I, partial deletion, and 1000 bootstrap replication. The tree was finally visualized using an interactive Tree of Life (iTol) online tool [38].

### 2.3. Chromosomal Location, Synteny Analysis, and Gene Duplication

The chromosomal information and distribution of the ramie WOX genes on chromosomes were obtained from genome data using TBtools. The genes were mapped to their chromosomal locations using Circos [39]. The genes’ segmental and tandem duplication events and the collinearity analysis between the ramie WOX genes and those from other representative species were performed using MCscanX [40]. The results were visualized using TBtools plugin for Advance Circos and multiple synteny. The rates of nonsynonymous and synonymous mutations were computed based on the coding sequence alignment using the KaKs calculator with the model average and averaging methods [41].

### 2.4. Gene Structure and Motif Analysis

The exon-intron arrangement of the ramie WOX genes and that of two other reference species were obtained using TBtools. Multiple Em for Motif Elicitation (MEME) online tools [42] version 5.4.1 was used to investigate the motifs of the WOX genes, with the parameters set as 20 maximum numbers of motifs and 6–200 optimum width of the motifs. The phylogenetic tree, exon-intron gene arrangement, and motifs were visualized in TBtools.

### 2.5. Expression Patterns Analysis

The potential involvement of the ramie WOX genes in adventitious root formation was investigated using quantitative real-time PCR and transcriptomic analysis of the ramie tissues grown using stem cuttings according to previous reports [43,44]. The transcriptome analysis of tissue-specific expression of the WOX genes was performed using four different ramie tissues (bud, leaf, stem, and root) sequenced by our laboratory, which was previously reported by Chen et al. [3].

Briefly, Zhongzhu No. 2 ramie variety seedlings were grown in earthen pots in a greenhouse at 25 ± 1 °C, 5000 lux light intensity, 75% ± 1 RH, with a 12 h photoperiod. Uniform-sized seedlings with similar characteristics were selected for cutting propagation. Tender stem cuttings 10–15 cm long with 2–3 leaves were grown under a hydroponics system and allowed seven days to acclimatize. At the onset of rooting sprouting, leaf samples from three independent biological replicates were collected for preliminary analysis using qRT-PCR. RNA was extracted and cDNA reverse transcribed using SteadyPure Plant RNA Extraction kit and Evo M-MLV One-Step RT-PCR Kit (Accurate Biotechnology Co. Ltd., Changsha, China) according to the manufacturer’s instructions. qRT-PCR was carried out on CFX96 Touch Deep Well Real-Time Quantitative PCR System (Bio-Rad) using SYBR^®^ Green Premix Pro Taq HS qPCR Kit II (Accurate Biotechnology Co. Ltd., Changsha, China) and gene-specific primers (Table S2). Relative expression levels were calculated using the 2^−∆∆Ct^, and the result was displayed in the form of a histogram drawn with the aid of GraphPad Prism v8.0 software. The data were illustrated as mean ± SD (standard deviation). The 18s gene (accession number EU747115) was used as reference internal control.

Samples for transcriptome analysis were collected 20 days after the culture and immediately frozen in liquid nitrogen [3]. Total RNA was extracted using TRIzol Reagent and Plant RNA Purification reagent (Invitrogen, Carlsbad, CA, USA) following the manufacturer’s procedure. The RNA integrity and concentration were checked using an Agilent 2100 bioanalyzer (Agilent, Santa Clara, CA, USA) and a NanoDrop 2000 spectrophotometer (Thermo Fisher Scientific, Waltham, MA, USA). The cDNA library was constructed with an Illumina Paired-end Sample Prep kit (Illumina, San Diego, CA, USA), quantified using TBS380 (Picogreen, Invitrogen, Carlsbad, CA, USA), and sequencing performed on the Illumina HiSeqTM2500 (2 × 150 bp read length) platform at Majorbio Bio-pharm Technology Co. Ltd., Shanghai, China.

The clean reads were mapped to the ramie reference genome. Gene expression levels were normalized as fragment per kilobase exon model per million mapped fragments (FPKM) and calculated using RSEM [45]. A heat map using the FPKM values (Table S3) was constructed [46,47] with TBtools. All the analysis was conducted using three biological replicates.

### 2.6. Adventitious Root Induction and Phenotypic Data

According to the previous method [2], tender stem cuttings (12–13 cm long) with 2–3 leaves of 67 ramie elite genotypes were grown under a hydroponics system. The cuttings were cultured using distilled water to induce adventitious roots, and the plants were maintained under a controlled environment (temperature of 25/30 °C, 60% relative humidity, and 14/10 h light/dark photoperiod) at the Chinese Academy of Agricultural Sciences Institute of Bast Fiber Crops. The number of root primordium for six biological replicates was recorded at 3 and 5 days post-culture. Root lengths for six biological replicates were measured using a meter rule at 15 days post-culture. The results were expressed as mean ± SD. Roots of 10 selected cuttings with significant variation in the number and length of root primordium were sampled for qRT-PCR analysis. The root samples were immediately frozen in liquid nitrogen. RNA extraction, cDNA synthesis, and expression analysis were carried out as described in Section 2.5 above. Three biological replicates were used for the analysis, and the data were illustrated as mean ± SD. The 18s gene was also used as a reference internal control.

### 2.7. Cis-Acting Element Analysis of the BnWOX14 in Ramie Elite Genetic Resources

The 4000 bp sequences upstream of the start codon of the ramie WOX gene *BnWOX14* from the ten elite genotypes selected were submitted to the PlantCare database to determine the cis-acting elements. The elements were classified into light-responsive, phytohormone-responsive, growth and development, as well as stress-responsive.

## 3. Results

### 3.1. Identification of Ramie WOX Genes

The genome sequence data of ramie was used to identify the WOX genes using BLAST search with the 14 *A. thaliana* WOX genes, resulting in a total of 17 candidate genes. The obtained sequences were further checked for the presence of homeodomain using the Conserve Domain Database and confirmed using Pfam and SMART. The genes were renamed following the methods reported by Wang et al. [23] as *BnWUS*, *BnWOX1A*, *BnWOX2 to BnWOX14* (Table 1). The number of amino acids and molecular weight of the BnWOX genes were in the ranges 123–856 and 14.38–93.65 kDa, respectively, with the lowest in *BnWUS* and the highest in *BnWOX10*. The isoelectric point (pI) was within the range of 4.91 (*BnWOX13A*) and 10.00 (*BnWUS*), with an average of 7.66. The highest grand average of hydropathicity (GRAVY) was obtained in *BnWOX10* (−0.115) and the least in *BnWUS* (−1.395). All 17 ramie WOX were predicted to be localized in the nuclear region (Table 1). However, *BnWOX10* and *BnWOX14*, in addition, are also indicated in the plasma membrane. The GRAVY result suggests that the BnWOX genes may be hydrophilic [23,48].

### 3.2. Multiple Sequence Alignment and Phylogenetic Analysis

Multiple sequence alignment and WebLogo were conducted using the 17 ramie WOX amino acid sequences and those from *A. thaliana* (14) and rice (13) to investigate the homologous domain and the degree of residue conservation in the domain. The distributions of the amino acid residues were very similar in all the three species (Figure S1A,B). Many highly conserved residues that comprise I, N, V, F, Y, W, Q, and R were found in helix 3, consistent with the literature [25,49]. Also, in helix 1, some residues like P, Q, and L were highly conserved. In helix 2, P and I were highly conserved. The highly conserved nature of the HB domains reflects the typical characteristics of WOX genes in both dicots and monocots [50].

**Figure 1:**
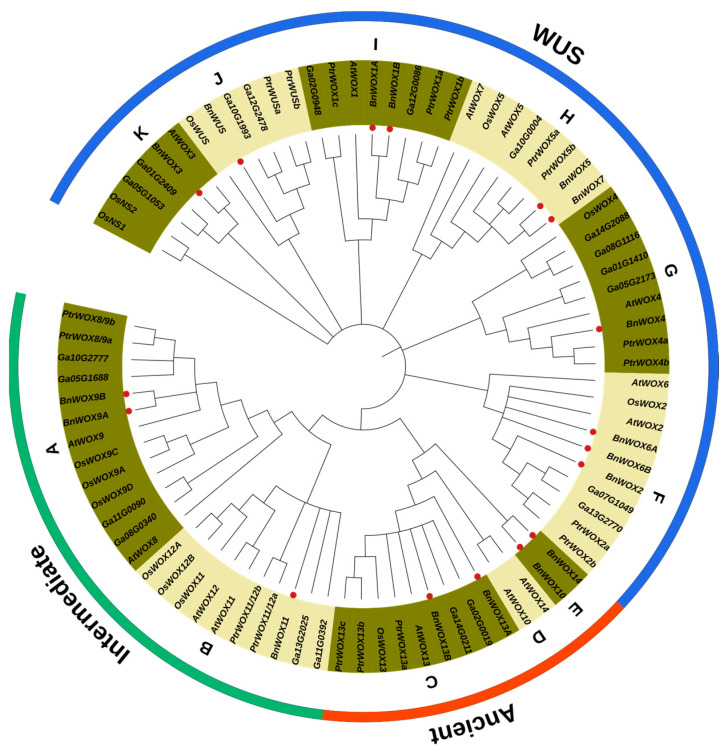
Neighbor-joining phylogenetic tree of the identified ramie WOX genes and those from *A. thaliana* and rice. The labels A to K indicate the subgrouping of the WOX genes, and the three groups marked ancient, intermediate, and WUS represent the members belonging to the specific clades.

Phylogenetic analysis was computed using a total of 83 deduced amino acid proteins from five different plant species to explore the evolution of ramie WOX family genes. The phylogenetic tree was divided into three groups: WUS, intermediate, and ancient clades (Figure 1), consistent with the literature [15]. The groups were further divided into 11 subgroups (A, B to K) based on closed homologies, with each subgroup having at least a member from all the five species except D and E, which are unique to *A. thaliana* and ramie, respectively. The WUS or modern clade has the highest number of subfamilies (6), comprising *BnWUS*, *BnWOX1*, *2*, *3*, *4*, *5*, *6*, and *BnWOX7*(F-K). The intermediate clades have only two subgroups, A and B, with the former comprising *WOX8/9* and the latter having WOX11/12 from all five species.

### 3.3. Chromosomal Location

The chromosomal positions of the 17 ramie WOX genes are shown in Table 1 and Figure S2. The genome has 14 chromosomes (chr), and the BnWOX genes are distributed in only 8 chr (chr1, chr2, chr3, chr7, chr10, chr12, chr13, and chr14). One of the WOX genes (*BnWOX13A*) was not conclusively assigned to any of the chr as reported in *TaWOX8* [17], which called for further investigation to ascertain its chromosomal positioning. Except for chr2 and chr14, which have three (*BnWOX9A*, *BnWOX9B*, and *BnWOX14*) and one (*BnWOX11*) genes, respectively, all the other six have two genes each. Ramie WOX genes from the same subgroup like *BnWOX1A/1B* (chr10), *BnWOX2/3* (chr3), *BnWOX5/7* (chr1), and *BnWOX6A/6B* (chr7) were located on the same chromosome. In contrast, genes belonging to different subgroups and clades like *BnWOX4/10* (chr13), *BnWUS/13B* (chr12), and *BnWOX9A/9B/14* (chr2) were also located on the same chromosomes. These, however, followed similar patterns with the WOX genes reported in other species like *Medicago sativa Triticum aestivum* and *Hordeum vulgare* [17,24].

### 3.4. Gene Structure and Motif Analysis

**Figure 2:**
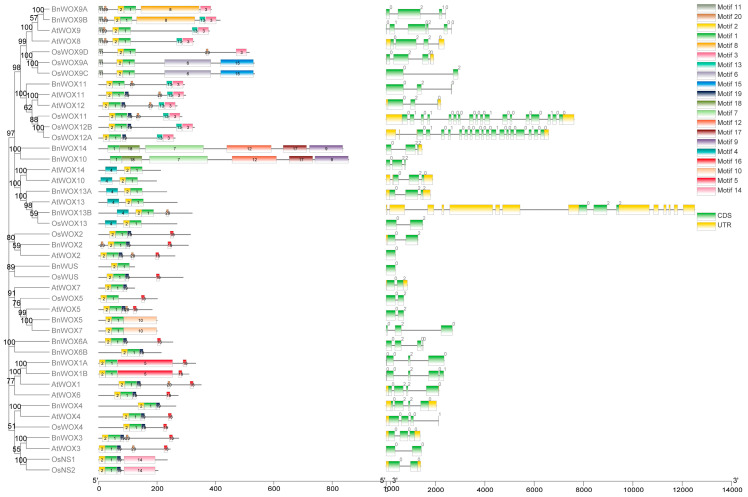
Gene structure and motif analysis of ramie WOX genes. A. Phylogenetic tree of the BnWOX genes and homologs from *A. thaliana* and rice. B. The gene motif classification shows 20 different motifs represented using various colors. C. Exon-intron structure of the WOX genes: the green box indicates the exon position, the line represents the intron, and the yellow box indicates the untranslated regions.

The presence of the HOX domain is the defining feature of the WOX gene family. A total of 20 motifs were found in the WOX genes, with the HOX domain motif1 present in all the BnWOX genes and their orthologues (Figure 2, Table S4). Motif2 is found in all the WOX genes except *BnWOX10* and *BnWOX14*. *BnWOX10/14*, however, uniquely have motifs 7, 9, 12, 17, and 18, which are lacking in all others. Some motifs were specific to only a clade. For example, motif16 was found in only members of the WUS clade, motif3 in only the intermediate clade members, and motif4 in the ancient clade. Others like motif5 (*BnWOX1A/1B*), motif8 (*BnWOX9A/9B*), and motif10 (*BnWOX5/7*) were present only in homologs genes, similar to what is obtained in rice *OsNS1/OsNS2* (motif14), and *OsWOX9A/9C* (motif6 and 15). The motif distribution supported the subgroup classification as members shared at least a common motif in addition to the HOX domain.

### 3.5. Gene Duplications and Synteny Analysis

The synteny analysis of the ramie WOX genes and those from rice and Arabidopsis (Figure S3) revealed the orthologues relationship between the genes. The result showed higher collinearity between the ramie and Arabidopsis, indicating a closer evolutionary relationship. Ramie and Arabidopsis have 11 ortholog gene pairs, against 6 between the former and rice. These conserved genes might have similar functions across different species [51]. Because gene duplication is crucial in the evolution process, gene functions, and plant diversity [52], we investigated the presence of segmental and tandem duplications in the orthologues gene pairs (Table S5). Four segmental duplications were obtained among the ramie WOX genes *BnWOX10/14*, *BnWOX13A/13B*, *BnWOX9A/9B*, and *BnWOX6A/Maker00021031*, which was segmentally duplicated with a different gene family member. All the remaining genes were singletons, except *BnWOX5/7*, which are tandemly duplicated pairs. Duplication leading to loss of function is permissible, as a single duplicate is required to maintain function, leaving the other under purifying selection. Gain of function results in acquiring a new advantageous allele. However, this rarely occurs in a population. Since gene duplicates are functionally redundant at the time of origin and usually nonfunctionalized [53], we determined the selection pressure among the ramie WOX genes duplicated pairs.

The rate of the nonsynonymous (Ka) and synonymous (Ks) substitutions among the segmentally duplicated pairs ranged from 0.0361 (*BnWOX10/14*) to 1.6615 (*BnWOX6A/Maker00021031*). The substitution rates were defined as Ka/Ks ˃ 1 (positive or diversifying selection), ˂1 (negative or purifying selection), and Ka/Ks = 1 as neutral mutation [51]. From the result (Table S5), we can deduce that all the duplicated genes have undergone purifying selection within the WOX family, except for *BnWOX6A* and its homolog that went through diversifying selection, which may be expected, as the homolog is not a WOX gene. Negative selection is essential in genome conservation and preservation of gene functions [52]. The overall result suggested duplication event as the significant driver of WOX genes in ramie, and the genes were under purifying selection.

### 3.6. Expression Pattern Analysis of the BnWOX Genes in Ramie Stem Cuttings of Hydroponic Elite Cultivar

**Figure 3:**
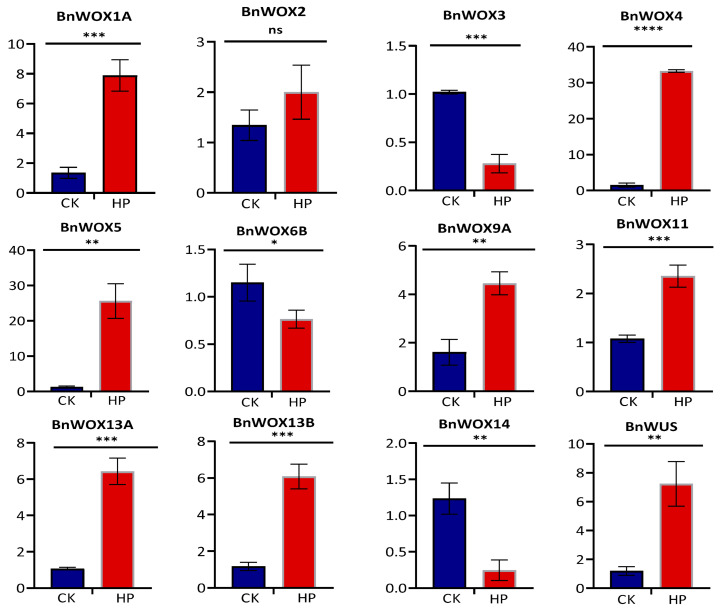
Relative expression analysis of the ramie WOX genes in ramie leaf stem cuttings. Data are means ± standard error of means (SE). HP represents the hydroponically grown stem cutting, and CK is the potted seedlings control. *, **, ***, **** indicate significant differences at *p* < 0.05, 0.01, 0.001 and 0.0001, respectively, whereas ns indicates no significant difference.

Adventitious root formation is vital in the vegetative propagation of the elite genotype, and studies have shown it to be a typical quantitative genetic trait [6]. Thus, we performed qRT-PCR analysis to investigate the WOX involvement in adventitious rooting of ramie cuttings. Due to the difficulty distinguishing the whole genome duplicated pairs because of the higher sequence similarities, only twelve genes out of 17 were tested. The result revealed that all the genes were upregulated in the leaf of the cuttings except for *BnWOX3*, *BnWOX6B*, and *BnWOX14*, which were downregulated (Figure 3). The overall result indicated all the genes have a potential role during the rooting of the cuttings and may also be involved in adventitious root formation.

**Figure 4:**
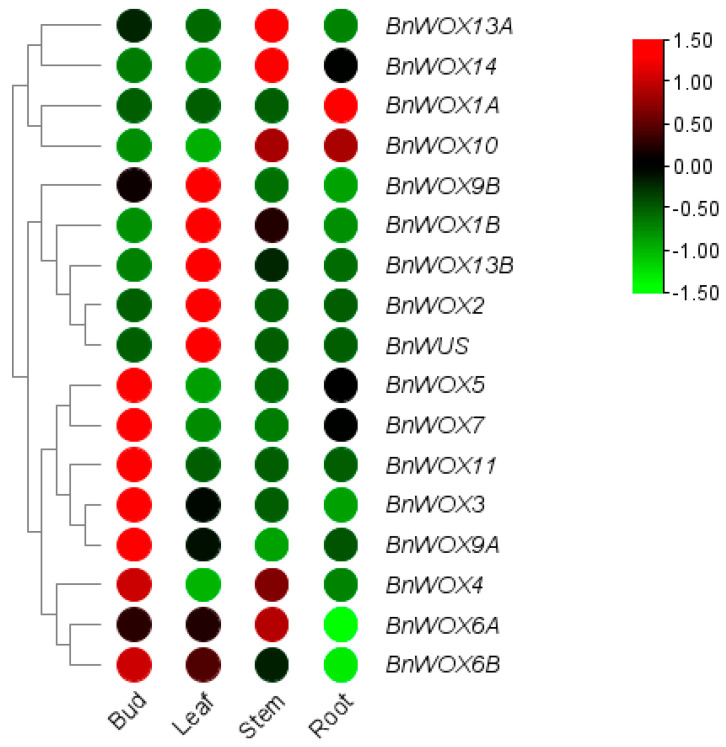
Expression profile of the ramie WOX genes in bud, leaf, stem, and root tissues using RNA-seq data. The scale (−1.50 to 1.50) represents the gene expression levels. The green indicates a low expression, whereas the red indicates higher expression.

Due to the close links of gene expression to its biological function [50], we analyzed the expressions of the genes in various tissues comprising the bud, leaf, stem, and root of the hydroponic elite ramie cultivar Zhongzhu No. 2 (Figure 4). All the genes showed a higher expression level in at least one of the four tissues. The majority of the genes displayed tissue-specific expression. Seven genes comprising *BnWOX3/4/5/6B/7/9A* and *BnWOX11* displayed the highest expression level in the bud. *BnWOX1A* had a more pronounced expression in only the root, while *BnWOX2* and *BnWUS* showed a significant expression in only the leaf. Most paralogous pairs with higher sequence similarity showed similar considerable expression levels in various tissues such as *BnWOX/5/7* (bud and root), *BnWOX6A/6B* (bud, leaf, and stem), and *BnWOX10/14* (stem and root) indicating similar biological functions. The expression of some of these genes in more than one tissue pointed to their roles in the development of the various organs.

Due to the highly conserved nature and functional diversity of WOX family subgroup members [15,54], the possible species-specific function of the *BnWOX10/14* as obtained from the phylogenetic analysis coupled with being the only two with significantly higher expression in both roots and stem, we proposed that they may be the primary genes regulating adventitious rooting in ramie. Interestingly, *BnWOX10* and *BnWOX14* were segmentally duplicated pairs with higher sequence homologies. The higher sequence similarities between the two WOX genes presented some challenges in getting a qRT-PCR primer specific to only *BnWOX10*, and, ultimately, we narrowed our further investigation to *BnWOX14*.

### 3.7. Expression of BnWOX14 and Adventitious Root Development in Different Ramie Genetic Resource

**Figure 5:**
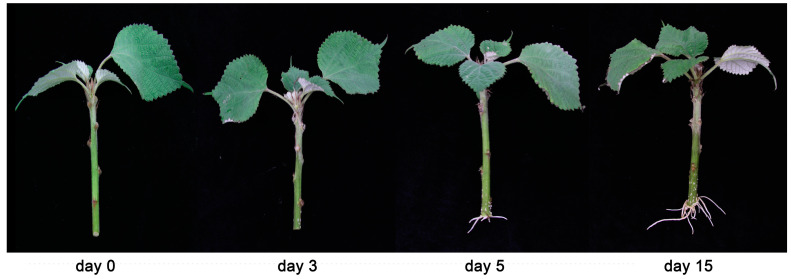
The development of adventitious roots in ramie cutting. The panel shows the rooting characteristics of Zhongzhu No. 2 as a representative. Stem cuttings of the varieties were hydroponically cultured for 20 days.

Because variability in plant species is critical and forms the basis for breeding targeted at enhancing adaptation, quality, and yield of produce [55], we investigate the rooting of ramie stem cuttings of 67 different genetic resources (Figure 5, Table S6) and expression analysis of the candidate to understand its functional role in adventitious root formation. The phenotypic rooting characteristics of the different resources showed remarkable variation, with most of the cuttings starting to produce roots after three days of the culture, while a few withered up.

Table 2 shows the resources’ top five, bottom five, and their rooting characteristics. Zhongzhu No. 1 had the highest number of root primordium five days post-cutting culture (12) and longest roots post-15 days (8.56 cm), followed by Zhongzhu No. 2, while Zhongzhu No. 3 and Guangxi-1 had the least of all the parameters measured.

**Figure 6:**
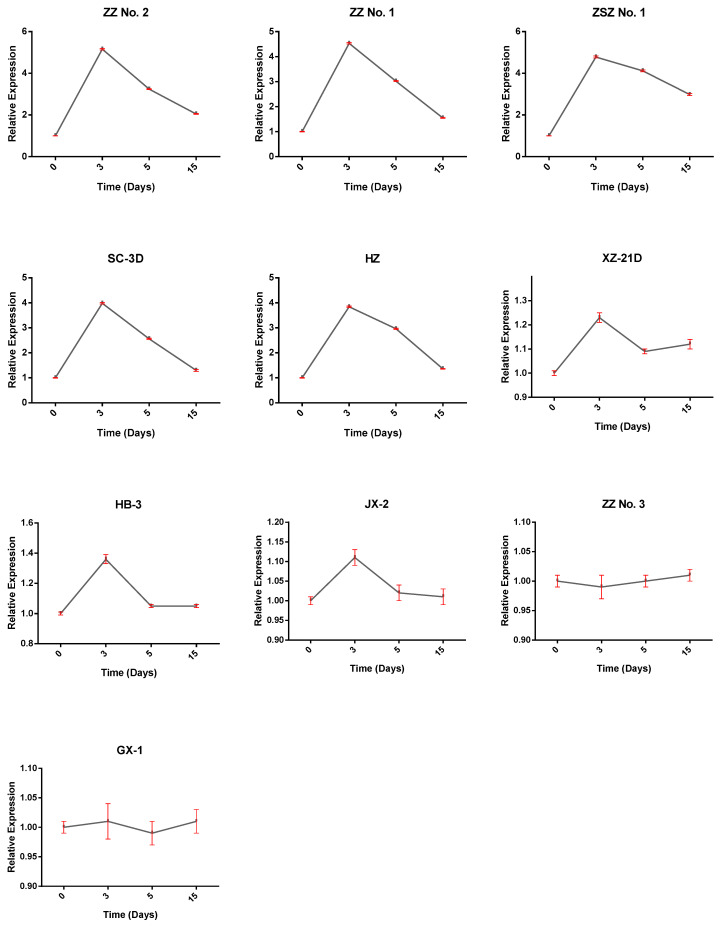
Relative expression of BnWOX14 in the roots of the stem cuttings of 10 different ramie genetic resources grown under hydroponics system. ZZ No. 2 = Zhongzhu No. 2; ZZ No. 1 = Zhongzhu No. 1; ZSZ No. 1 = Zhongshizhu No. 1; SC-3D = Sichuan-3D; HZ = Huazhu; XZ-21D = Xiangzhu-21D; HB-3 = Hubei-3; JX-2 = Jiangxi-2; ZZ No. 3 = Zhongzhu No. 3; GX-1 = Guangxi-1.

Expression of *BnWOX14* showed a variation between the cuttings with the longest adventitious root and those with the lowest. The top five genetic resources (Zhongzhu No. 2, Zhongzhu No. 1, Zhongshizhu No. 1, Sichuan-3D, and Huazhu, XZ-21D) showed similar expression patterns. Their expression at 3, 5, and 15 days post-culture was remarkably higher than in all the bottom five resources (Figure 6). Although some variation exists among the bottom five, the relative expressions were comparably low. The overall result demonstrates the potential function of *BnWOX14* in regulating adventitious rooting in ramie.

### 3.8. Cis-Acting Element Analysis of the BnWOX14 in the Elite Genetic Resources

Cis-acting elements found upstream of the coding sequence of genes regulate gene expression by interacting with the transcription factors [24]. The *BnWOX14* cis-elements in the promoter of the ten ramie genetic resources were categorized into four prominent groups: light-responsive, phytohormone-responsive, growth and development, and stress-responsive (Table S7). The most prominent elements in the different resources were GT1 (light-responsive), ABRE (phytohormone-responsive), and ARE and WUN (stress-responsive). ABRE is an abscisic acid-responsive element that regulates abscisic acid-induced gene expression. ARE is an anaerobic-responsive element that induces an anaerobic response, and WUN is a wound-responsive element. Though there was no significant variation in the number of elements across the different genetic resources, there is a difference in their position, which may affect their regulatory function. A space as little as 2 bp in GT1 of the rbcS-3A in pea showed a dramatic reduction in transcript level in vivo, as did deletion of 3 or 8 bp [56]. A deletion of 10 bp was also found to have resulted in GT1 loss of activity.

## 4. Discussion

Adventitious roots are formed from the non-root pericycle cells of leaves, stems, or older roots, and they provide a critical base for clonal propagation of numerous crop and forestry plants [57]. Adventitious root formation is regulated by an extensive network of transcription factors, such as the WOX proteins [58]. Others include the *AUXIN RESPONSE FACTOR* (ARF), *APETALA2/ETHYLENE RESPONSE FACTOR* (AP2/ERF) like *AINTEGUMENTA-LIKE* (AIL), and the GRAS family like the *SHORTROOT* (SHR); the *SCARECROW* (SCR); and the *SCARECROW-LIKE* (SCL) proteins [59]. The WOX gene forms a large transcription factor family essential in the maintenance of stem cells, embryo patterning, and the formation of lateral organs. The roles of reverse and forward genetics were recently revealed in many developmental and physiological processes [25,50]. Identification and understanding of the roles of WOX genes in controlling adventitious rooting in ramie will enhance productivity.

Here, we identified 17 ramie WOX genes using genome-wide analysis. Variable numbers of WOX genes were reported from different plant species, such as 14 in rice, 12 in poplar, 19 in maize, 11 in cacao, and 21 in *Gossypium arboreum*, and the numbers of the WOX gene family members are independent of genome size [33,34,50]. Following phylogenetic analysis, the genes were grouped into three classical groups (WUS/modern, intermediate, and ancient). The modern clade contains six subgroups, while the intermediate and ancient have two and three subgroups, respectively. Consistent with this result, the modern clade was reported to have the highest number of subgroups/subfamilies in other plant species such as apple, wheat, rice, maize, etc. [17,34], indicating the reliability of the data and highly conserved nature of the HB across plants. In addition, all the ramie WOXs were predicted to be confined to the nucleus. In line with our findings, all the WOX genes identified in *Phalaenopsis equestris* (14 PeWOX) and *Dendrobium catenatum* (10 DcWOX) were predicted to be localized in the nuclear regions except *DcWOX9*, which has either the chloroplast or extracellular region as the potential subcellular location [48]. Transient expression assay of the *A. thaliana* WUS gene fused with the reporter gene glucuronidase (GUS) confined the WUS subcellular location to the nucleus. Similarly, rice *WOX11* fused with the green fluorescent protein (GFP) also confined the gene location to the nucleus [60].

Since WOXs are evolutionarily conserved in plants [61], members in the same monophyletic clade may have similar functions. *AtWOX3* and rice homolog *OsNS1/OsNS2* play a role in recruiting founder cells from the shoot apical meristem lateral domain to form marginal and lateral leaf regions, which subsequently divide to form leaf buttresses, and finally the adaxial–abaxial axis [62,63]. The functional conservation of these genes suggests that *BnWOX3* may also play a similar role in forming the adaxial–abaxial axis in ramie. WOX5/7 functions during the transition of root founder cells to the root primordium and are considered a potential marker in the root primordium [64]. Interestingly, all the WOX5/7 from the five species belong to the same subgroup (H), suggesting that *BnWOX5/7* may also be a marker in root primordium. The intermediate clades have only two subgroups, with members exclusively WOX8/9 (A) and WOX11/12B. The two subgroups have a reference function in regulating primary root and adventitious founder cells [13]. Overexpression of orthologs of *BnWOX11* (*AtWOX11/12*, *OsWOX11*, *PeWOX11a*/*b*) was found to have increased the number of adventitious roots and root biomass in Arabidopsis, rice, and poplar, suggesting that *BnWOX11* may be involved in increasing root biomass in ramie [13,58,60]. *AtWOX9* and its orthologs are involved in vegetative shoot apical meristem growth and embryogenesis [18,65,66], suggesting that *BnWOX9A/9B* may have similar functions in embryogenesis.

The ancient clade, which characteristically has WOX10, WOX13, and WOX14 as members, is subdivided into three subgroups. Though WOX13 of all the five species belong to a unique subfamily (C), *AtWOX10/AtWOX14* (D) and *BnWOX10/BnWOX14* (E) belong to different subfamilies. *AtWOX13* and *AtWOX14* are expressed in the floral organs and the primary and lateral roots, with a possible role in preventing premature differentiation [15]. These suggested that *BnWOX10/14* may also play a role in root architecture and promote abiotic stress tolerance [67].

Because gene structure is closely related to gene function, it was investigated to understand the ramie WOX genes better. All the genes, including those from *A. thaliana* and rice, have at least one intron, except *BnWUS* and its rice orthologue (*OsWUS*). Most genes have either two or three exons. Orthologous genes and those in the same clade generally have a similar exon-intron arrangement consistent with the phylogenetic relationship. WUS box, [EQK]TL[EQ]LFP[LV][QR][PS]T[GN] is found to be present in almost all the WUS clade members. The WUS box and the presence of acidic regions in the WOX genes aid their functions as transcription factors [25,68].

Genome duplication may have impacted the number of WOX genes in ramie, as four segmental duplications and one tandem were found. We observed the expansion event of *BnWOX5/7* and *BnWOX6A/Maker00021031* in the modern clade, *BnWOX9A/B* in the intermediate clade, and *BnWOX13A/B* and *BnWOX10/14* in the ancient clade. The Ka/Ks analysis indicated all but *BnWOX6A* to have undergone purifying selection. The duplication events are vital in understanding the mechanisms of genetic redundancy, which often lead to the loss of functions (non-functionalization), the gain (neo-functionalization), or the partition (sub-functionalization) of the original gene(s) functions. When such duplicated genes are preserved via sub-functionalization, they may come under different selective pressures relative to their shared ancestor [69]. Some duplicated pairs like *BnWOX10/14* have shown similar transcript expression levels, and some have undergone speciation, such as *BnWOX13A*, with a higher transcript in the stem in contrast to *BnWOX13B*, having a higher transcript in the leaf. *BnWOX9A* and *BnWOX9B* similarly showed speciation, with the former having a higher transcript level in the bud and the latter in the leaf. These suggested that the two segmental duplicated pairs (*BnWOX13A/13B* or *BnWOX9A/9B*) might have gained a new regulatory role, leading to either neo-functionalization or sub-functionalization. Nevertheless, *BnWOX9A/9B* displayed unique motif8 absent in all others, as *BnWOX10/14* also lacked motif2 found in all others.

Expression profile analysis of the BnWOX genes in the bud, leaf, stem, and root of the hydroponic cultured stem cutting displayed a tissue-specific expression. *BnWOX10* and *BnWOX14* had comparably higher expression levels in the stem and the root and lower in the leaf (consistent with qRT-PCR result). As a potential adventitious root regulator, higher activity is expected in the stem and the adventitious root. Their orthologs from rice and *Arabidopsis* have been reported to regulate root architecture, promote vascular tissues, and stimulate gibberellins’ anabolism [70,71]. Mutation upstream of the *AtWOX14* resulted in abnormal root development and delayed floral transition [15]. Since *BnWOX10* and *BnWOX14* represent the most conserved and functionally diverse subgroup members [15,54], close homologies to *AtWOX10/14*, and higher expression levels in the adventitious root and stem, we opined that they might have a critical role in adventitious rooting in ramie. Coincidentally, the two are segmentally duplicated pairs; we subsequently investigated the expression pattern of the *BnWOX14* in different ramie genetic resources and cis-acting elements in their specific promoters.

Analysis of the ramie *BnWOX14* promoters of various genetic resources revealed several cis-acting elements, including the auxin-responsive motif (TGA-element), the methyl jasmonate-responsive element (TGACG/CGTCA), and ERF. Plant hormones are a key internal factor controlling adventitious root formation, with auxin being central [72,73]. In addition, ethylene is reported as a stimulant of adventitious roots during the early induction and late formation phases and positively correlates with the number of adventitious roots. Several ethylene-responsive elements (ERFs) were also found to have been continuously upregulated during adventitious root formation in ramie [57]. Recent findings also reported the positive role of jasmonic acid in the adventitious rooting of cuttings from petunia and pea, independent of auxin, ethylene, or carbohydrate metabolism [57,74,75].

In addition, all the resources have one or more stress-responsive cis-acting elements, such as ARE and WUN, indicating possible roles in regulating stress responses. However, the MYB binding site involved in drought inducibility (MBS) was found in only two of the resources. This agrees with the assertion that elements like MBS have selectively evolved due to environmental pressure [24]. Generally, all the resources have comparably the same number of elements, which varies with position. We proposed that the difference recorded in the adventitious rooting and expression of the *BnWOX14* in the ten genotypic resources might be due to different cis-acting element positions, which have been confirmed as significant players in the function of genes [56].

## 5. Conclusions

The WUSCEL-related homeobox gene family is a crucial regulatory gene family involved in adventitious root formation and stem cell function. In this study, we identified 17 WOX genes in ramie and evaluated their structure, chromosome location, duplications, and expression profile. We also performed expression analysis of the genes in the stem, leaf, bud, and stem cuttings’ roots. Based on the analysis, we suggested *BnWOX14* as crucial in the species’ adventitious rooting; we further evaluated its expression in 67 different ramie genetic resources. This study provides insight into the characteristics and potential functions of the WOX gene family in ramie and will contribute to future research.

## Figures and Tables

**Table 1:** Physical and chemical characteristics of WOX genes identified in ramie.

Gene Name	Gene ID	Chromosome Position	No. A. Acids	M.wt. (Da)	*pI*	GRAVY	Subcellular Location
*BnWOX1A*	Maker00062999	Chr10:2590680-2593026	332	37,374	8.16	−0.956	Nuclear
*BnWOX1B*	Maker00074103	Chr:2485054-2487384	309	34,964	8.85	−1.032	Nuclear
*BnWOX2*	Maker00030742	Chr3: 7583477-7584960	307	34,320	6.70	−0.825	Nuclear
*BnWOX3*	Maker00031089	Chr3: 8055078-8056503	274	31,317	8.42	−0.942	Nuclear
*BnWOX4*	Maker00002967	Chr13: 2041939-2044060	264	29,331	9.54	−0.826	Nuclear
*BnWOX5*	Maker00093460	Chr1:16210128-16210838	200	22,959	8.56	−0.714	Nuclear
*BnWOX6A*	Maker00053871	Chr7: 3319544-3322227	254	28,885	8.58	−0.525	Nuclear
*BnWOX6B*	Maker00021009	Chr7: 1475052-1476549	214	24,453	9.80	−0.658	Nuclear
*BnWOX7*	Maker00093478	Chr1: 16182683-16183394	200	22,984	8.78	−0.746	Nuclear
*BnWOX9A*	Maker00080289	Chr2: 4246373-4248777	386	42,823	7.15	−0.746	Nuclear
*BnWOX9B*	Maker00047861	Chr2: 7182597-7185242	416	45,763	7.81	−0.595	Nuclear
*BnWOX10*	Maker00082428	Chr13: 9054047-906063	856	93,650	5.80	−0.115	Nuclear/Plasma membrane
*BnWOX11*	Maker00086436	Chr14: 2642861-2645770	294	32,603	5.67	−0.452	Nuclear
*BnWOX13A*	Maker00063698	Contig314_1: 496112-497997	233	26,515	4.91	−0.819	Nuclear
*BnWOX13B*	Maker00008406	Chr12: 12559425-12571929	320	36,059	5.56	−0.953	Nuclear
*BnWOX14*	Maker00091405	Chr2: 18352039-18359653	837	91,817	6.03	−0.119	Nuclear/Plasma membrane
*BnWUS*	Maker00008321	Chr12: 11915820-11916191	123	14,384	10.00	−1.395	Nuclear

**Table 2:** Phenotypic statistics of adventitious root development in the ramie resources.

	Germination of Root Primordium	Development of Adventitious Roots	
	Average Number of Primordium after 3 Days	Average Number of Primordium after 5 Days	Average Length (cm) after 15 Days
ZZ No. 2	3 ± 0.83 ^b^	12 ± 1.48 ^a^	8.56 ± 1.09 ^a^
ZZ No. 1	1 ± 0.45 ^c^	10 ± 1.22 ^b^	6.22 ± 1.13 ^b^
ZSZ No. 1	5 ± 0.98 ^a^	7 ± 1.22 ^c^	4.74 ± 0.55 ^c^
SC-3D	3 ± 0.00 ^b^	7 ± 1.96 ^c^	4.25 ± 0.54 ^c^
HZ	1 ± 0.00 ^c^	4 ± 0.45 ^d^	3.98 ± 0.44 ^c^
XZ-21D	0	2 ± 0.00 ^f^	0.33 ± 0.01 ^d^
HB-3	0	2 ± 0.44 ^f^	0.33 ± 0.01 ^d^
JX-2	0	3 ± 0.44 ^e^	0.32 ± 0.01 ^d^
ZZ No. 3	0	3 ± 0.00 ^e^	0.30 ± 0.01 ^d^
GX-1	0	3 ± 1.09 ^e^	0.30 ± 0.01 ^d^

ZZ No. 2 = Zhongzhu No. 2; ZZ No. 1 = Zhongzhu No. 1; ZSZ No. 1 = Zhongshizhu No. 1; SC-3D = Sichuan-3D; HZ = Huazhu; XZ-21D = Xiangzhu-21D; HB-3 = Hubei-3; JX-2 = Jiangxi-2; ZZ No. 3 = Zhongzhu No. 3; GX-1= Guangxi-1. Means with different letters are statistically different (*p* < 0.05).

## Data Availability

Data are contained within this article and the supplementary files. Other materials/information may be requested from the corresponding authors.

## Supplementary Materials

The following supporting information can be downloaded at: https://www.mdpi.com/article/10.3390/biology12121475/s1, Figure S1: Multiple sequence alignment and WebLogo of ramie WOX genes; Figure S2: Chromosome positioning of ramie WOX genes; Figure S3: Synteny analysis of ramie WOX genes and those from Arabidopsis and rice; Table S1: Accession numbers of Arabidopsis and rice WOX genes used for the analysis; Table S2: qRT-PCR primers used for expression analysis of ramie WOX genes; Table S3: FPKM values of the WOX genes from normalized transcriptome data, Table S4: Motifs consensus sequences of ramie WOX genes; Table S5: Duplication events of ramie WOX genes; Table S6: Ramie genetic resources and rooting characteristics; Table S7: Cis-acting element in the promoter of *BnWOX14*.
