# Supplementary figures and images for: Comprehensive Analysis of WUSCEL-Related Homeobox Gene Family in Ramie (Boehmeria nivea) Indicates Its Potential Role in Adventitious Root Development
Source: Biology (Basel). 2023 Nov 28;12(12):1475. doi: 10.3390/biology12121475 (PMC10740585; doi:10.3390/biology12121475)

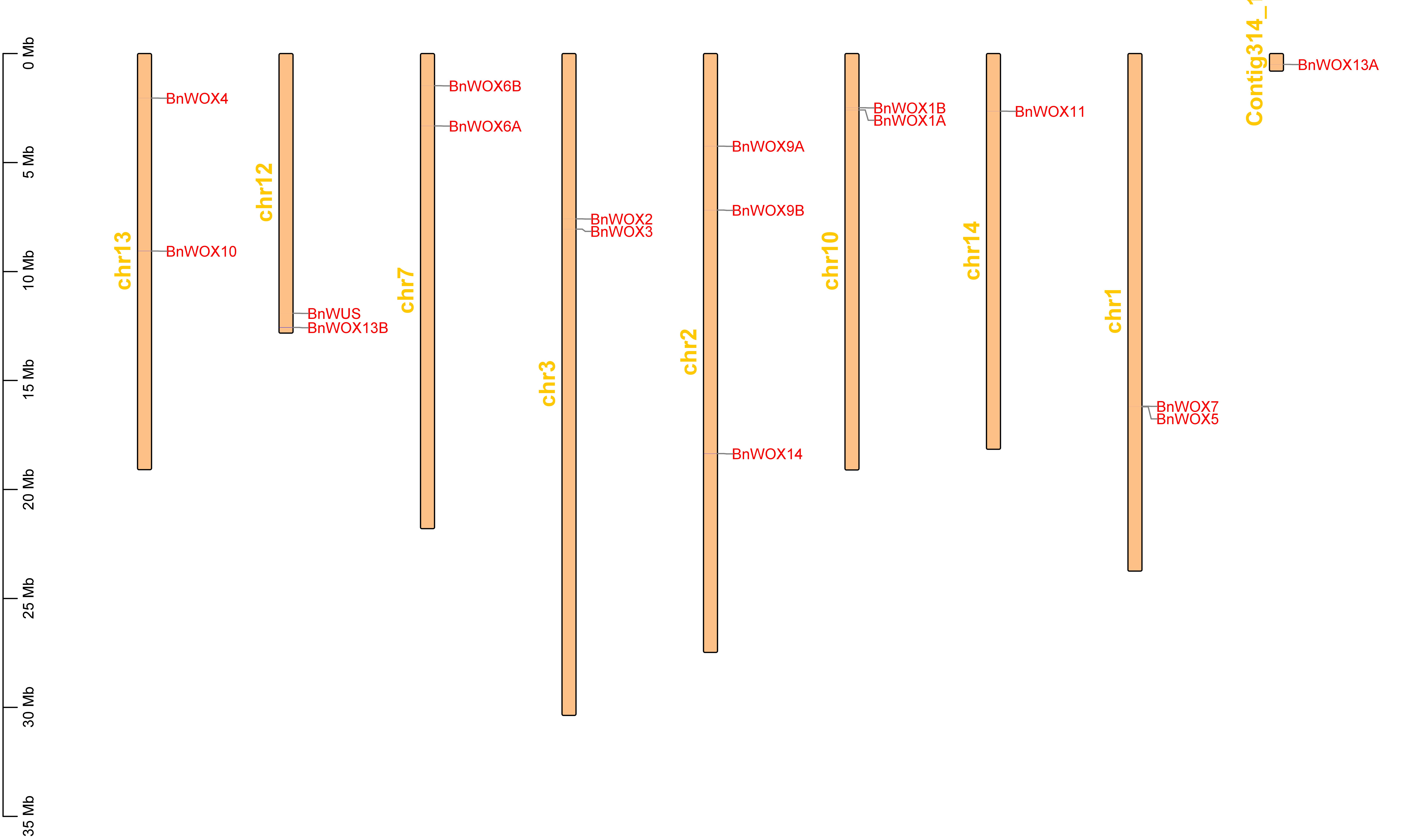

Supplement: Supplementary file 1 [file biology-12-01475-s001.zip › Figure S2_Chromosome location.jpg]

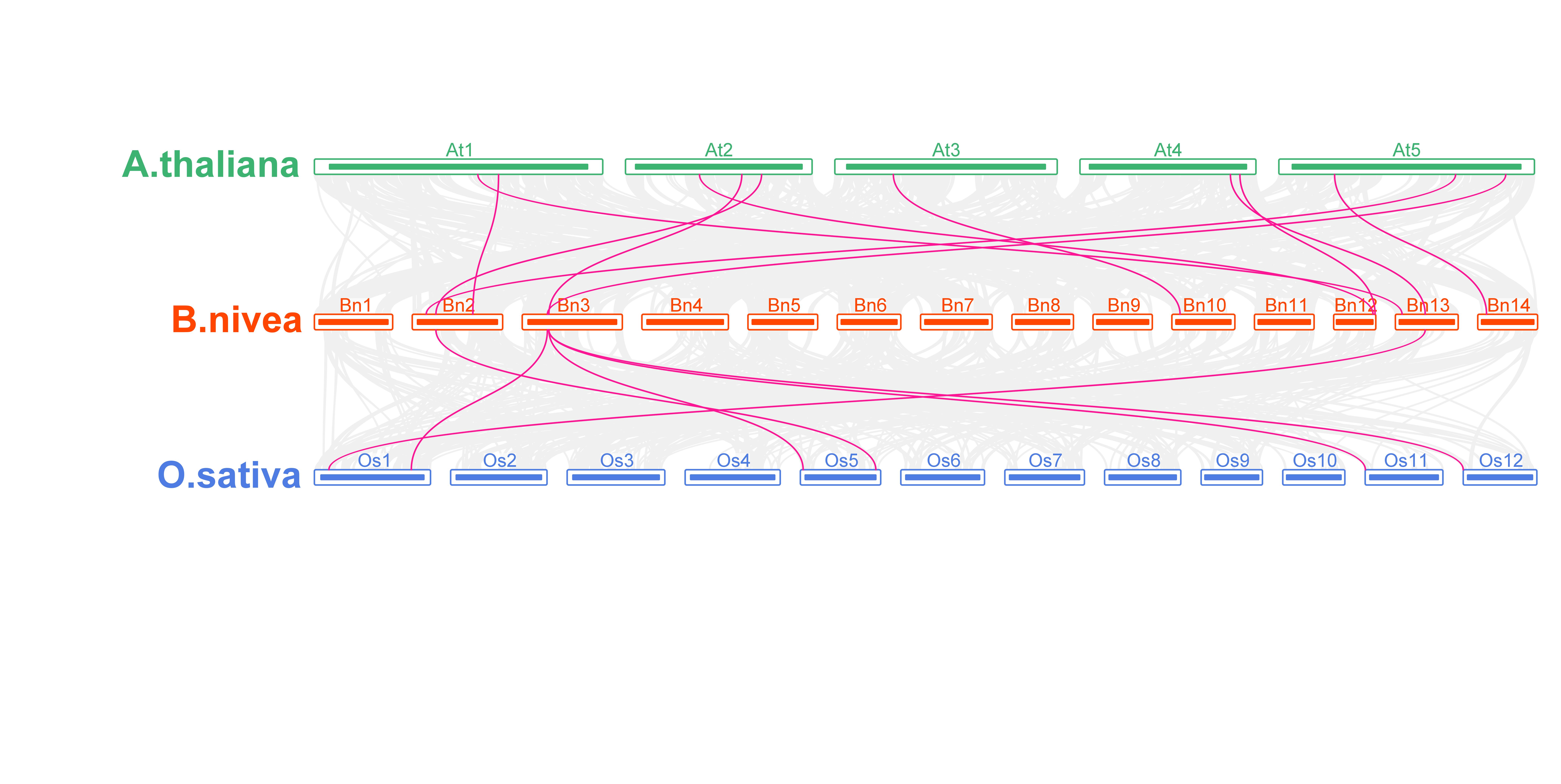

Supplement: Supplementary file 1 [file biology-12-01475-s001.zip › Figure S3 Synteny analysis.jpg]
